# Comparison of sagittal spinal alignment on standing plain x-rays and supine MRI in degenerative lumbar disease

**DOI:** 10.3389/fsurg.2023.1103952

**Published:** 2023-02-22

**Authors:** Xiaolong Fan, Deting Xue, Zhijun Pan, Yulu Wang

**Affiliations:** ^1^ Department of Orthopedics, 1st Affiliated Hospital of Baotou Medical College, Baotou, China; ^2^ Department of Orthopaedics, 2nd Affiliated Hospital, School of Medicine, Zhejiang University, Hangzhou, China

**Keywords:** degenerative lumbar disease, sagittal spinal alignment, standing plain x-rays, supine MRI, spine

## Abstract

**Background:**

The purpose of the present study is to examine the possible correlation between standing plain x-rays and supine magnetic resonance imaging (MRI) for evaluating spinal sagittal alignment in degenerative lumbar disease (DLD).

**Methods:**

The characteristics and images of 64 patients with DLD were reviewed retrospectively. The thoracolumbar junction kyphosis (TJK), lumbar lordosis (LL) and sacral slope (SS) were measured on lateral plain x-rays and by MRI. Inter- and intra-observer reliability was tested using intra-class correlation coefficients.

**Results:**

The results suggested that TJK measurements obtained from MRI tended to underestimate the radiographic measures by 2°, whereas SS measurements obtained from MRI tended to overestimate the radiographic measures by 2°. The LL measurements obtained from MRI were approximately equal to the radiographic measures, and the x-ray and MRI measurements were linearly related.

**Conclusions:**

In conclusion, supine MRI can be directly translated into sagittal alignment angle measurements obtained from standing x-rays with an acceptable degree of accuracy. This can avoid the impaired view caused by the overlapping ilium, while reducing the patient's exposure to radiation.

## Key points

• Supine MRI and standing x-ray measurements have a strong correlation
• Supine MRI can be used to evaluate sagittal alignment of the spine
• MRI measurement can be more accurate than x-ray in sagittal measurement

## Research significance

This study found that supine magnetic resonance imaging (MRI) can be used to assess the sagittal position of the spine without unnecessary radiation exposure. Upright x-ray allows for more accurate sagittal measurements that can be directly converted into sagittal alignment angles to ensure the accuracy of those obtained by x-ray. The use of MRI can avoid impaired visibility caused by iliac bone overlap and reduce patient exposure to radiation. The above findings provide a more accurate basis for the clinical diagnosis of degenerative lumbar diseases (DLD). Selecting appropriate imaging examinations according to different types of DLD can avoid unnecessary radiation exposure and economic burden and enrich the field of DLD research. By expanding the sample size of the test group and controlling the influence of confounding factors, the changes of lateral muscles, intervertebral discs and vertebral bodies in patients with different degrees of DLD will be better understood, as well as what imaging diagnosis is more accurate for different diseases.

## Introduction

Degenerative lumbar disease (DLD) refers to the physiological and pathological process of natural ageing and degeneration of the lumbar spine. Globally, 266 million people suffer from DLD each year; 390 million people experience lumbar spondylolisthesis each year, 403 million experience symptomatic disc degeneration and 103 million experience spinal stenosis (1). The current examination methods for DLD rely primarily on imaging. Magnetic resonance imaging (MRI) T2 weighted images can effectively show spinal canal / intervertebral foramen stenosis, and MRI short T1 inversion recovery sequence shows early spinal fracture and inflammation. Full spine x-rays help show spinal sequence, supine x-rays show lumbar instability (2). Spinal surgery is the primary treatment for DLD, and spinal alignment (including coronal and sagittal alignment) is an important aspect of preoperative planning and surgical reconstruction of the spine (3–6).

In DLD, coronal alignment reconstruction is an important factor in degenerative lumbar scoliosis, whereas sagittal alignment is usually considered in spondylolisthesis and loss of lumbar lordosis (LL) (7). The standing x-ray is considered the gold standard for measuring spinal alignment (8). However, repeated exposure of patients to high doses of radiation increases the risk of developing health problems (9). In addition, the lateral radiological assessment of the lumbosacral region is usually obscured by the overlapping ilium (10). On the other hand, using MRI, because of its high soft tissue resolution, the intervertebral disc, ligaments and other tissue can be clearly observed, along with the central spinal canal and nerve foramen; so, in patients with lumbar spinal stenosis, MRI examination has more advantages (11). Since MRI does not involve radiation, it is a favourable alternative to plain radiographs for obtaining images of the spine. However, it is performed in the supine position; consequently, the angle measurements are underestimated compared to standing x-rays due to the effects of gravity.

Several studies have examined the relationship between the images produced by standing plain x-rays and supine MRI images in determining spinal alignment (12–14). Wang et al. (12) found that supine axial loaded MRI correlated well with standing Cobb angle measurements. Sun et al. (13) demonstrated that unloaded spine MRI could reliably be translated into equivalent radiographic measurements with an acceptable range of error in adolescent idiopathic scoliosis. Baldairon et al. (14) found that supine MRI was a valid alternative to standing x-rays for measuring upper thoracic kyphosis in the sagittal plane. However, the efficacy of x-ray and MRI in evaluating spinal sagittal alignment in DLD is controversial (15, 16), and further studies are needed to evaluate their effectiveness.

Therefore, this study compared standing plain x-rays and supine MRI for evaluating spinal sagittal alignment in DLD and examined the possible correlation between the measurements obtained using the two imaging modes.

## Material and methods

### Study subjects

This study used a retrospective design and a convenience sampling method. It included patients who underwent standing film and MRI examinations and were diagnosed with DLD at this hospital between 1 January 2015 and 1 June 2016. The Institutional Review Board approved the study (approval number: ID:2014–426), and each patient gave written consent. The inclusion criteria were as follows: (1) patients with lumbar spinal stenosis, lumbar disc hernia or lumbar spondylolisthesis; (2) age ≥ 50 years; (3) no underlying congenital or neurological abnormalities; (4) no leg length discrepancy > 2 cm; (5) Cobb angle < 20°; (6) no more than 1 month between the standing x-ray and MRI. The exclusion criteria were as follows: (1) previous spinal surgery; (2) incomplete clinical and imaging data; (3) nondegenerative spine pathologies; (4) fracture of a vertebra. The standing radiographs were taken with a digital flat panel detector system, and the MRI was obtained with a 1.5 T MRI scanner (Siemens, Germany). All radiographic and MRI images were assessed by means of the picture archiving and communication system (PACS) workstation of our hospital.

### Study methods

**Figure 1:**
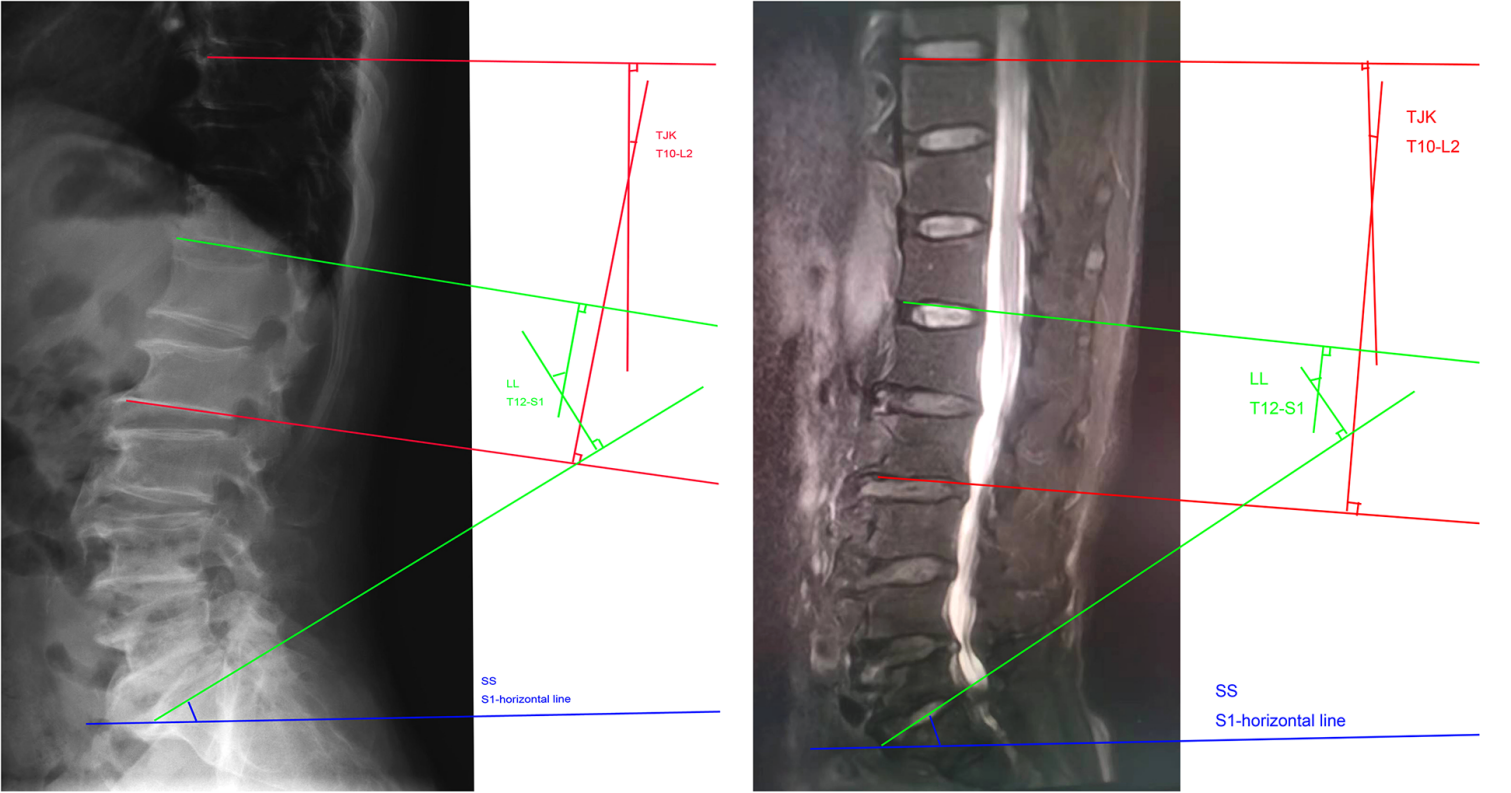
x-ray (side view) and MRI images with angle measurement marks. (**A**) X-ray imaging, (**B**): MRI imaging. MRI, magnetic resonance imaging.

The radiological examination protocol was standardised for all the participants. For each participant, standing anteroposterior and left lateral x-rays covering the spine and pelvis were obtained. The supine MRI covered the region from T10 to the coccyx. During MRI examination, the patient was placed in supine position with their legs flat. All lumbar images were acquired with a 3.0 T system (GE Signa HDx 3.0 T) using a digital posterior coil. The following scan parameters were used: slice thickness, 4 mm; repetition time (TR), 3000 ms; echo time (TE), 100 ms; matrix, 704 × 704; reconstructed voxel size, 0.43 × 0.43 × 4 mm; acquired voxel size, 0.60 × 0.86 × 4 mm; scan duration, 4:12 min. All lumbar x-rays were taken in a standing position using a digital tablet detection system. The following scan parameters were used: tube voltage, 90 kV; tube current, the average dose was 1.5 mSv. In the anterior lumbar spine x-ray, the position was supine, the arms were placed on the chest, and the hips and knees were flexed. The centreline entry point was 3 cm above the umbilicus (about the level of the third lumbar vertebra), and the film was ingested vertically on the bed surface. For the lumbar lateral x-ray, the position was side lying, the arms were flexed, the head was raised, the legs were together, the hips and knees were flexed and the coronal plane was perpendicular to the bed surface. The film was vertically ingested at the centreline, 3 cm upward through the highest point of the iliac spine, i.e., at the level of the third lumbar vertebra. The following lumbopelvic sagittal alignment parameters were assessed on both radiographs and MRI images: the thoracolumbar junction kyphosis (TJK) was measured from the superior end plate of T10 to the inferior end plate of L2; LL was measured as the subtended angle between tangents of T12 lower endplate and S1 sacral endplate (Figure 1); sacral slope (SS) was the angle between the superior end plate of S1 and a horizontal line. On MRI images (T1 weighted images) in supine position, angles were measured according to the standing radiographs. Only one midline-copied image was measured to ensure consistency.

A resident of the department who did not participate in the later analysis collected these cases from the database. Two independent expert spine surgeons, unaware of the identity of the patients and the treatment they received, were selected to separately assess the images and measurement parameters.

### Statistical analysis

Statistical analysis was carried out with Statistical Product and Service Solutions (SPSS) (version 22.0). Metric variables were descriptively reported using means (standard deviation). The interobserver reliability of the lumbopelvic parameters was tested by using intraclass correlation coefficient (ICC). The pairwise t-tests were used to evaluate the differences between radiographs and MRI images. The Pearson correlation coefficient was used to analyse the correlation in lumbopelvic parameters between the two imaging studies. *P*-values < 0.05 were considered statistically significant.

## Results

### The basic information of subjects

The study enrolled 64 patients [30 males, 34 females; average age, 62 ± 7 years; mean body mass index (BMI), 23.79 ± 3.1 kg/m^2^]. Thirty-three patients were diagnosed with lumbar stenosis; 20 patients with lumbar disc herniation; and 11 patients with lumbar spondylolisthesis (Table 1).

**Table 1:** Basic characteristics of included subjects.

Variable	Number (%)
**Gender**	
Man	30 (46.87)
Woman	34 (53.13)
**Age (year)**	62 ± 7
**BMI(kg/m^2^)**	23.79 ± 3.1
**Disease Type**	
Degenerative lumbar spinal stenosis	33 (51.56)
Lumbar disc herniation	20 (31.25)
Lumbar spondylolisthesis	11 (17.17)
**Total**	64 (100.00)

Note: BMI, body mass index.

### Comparison of MRI and x-ray parameters

The average time between the two imaging methods was 1 ± 1 week. The average TJK measurement from the x-ray and MRI images was 11 ± 9° and 9 ± 7°, respectively; the average LL was 40 ± 16° and 40 ± 13°; and the average SS was 33 ± 11° and 35 ± 9°. The differences were not statistically significant (*P *< 0.05). (Table 2).

**Table 2:** Comparison of X—ray and MRI parameters.

Parameter	x-ray	MRI	*t*	*P* value
TJK	11 ± 9°	9 ± 7°	0.473	0.647
LL	40 ± 16°	40 ± 13°	0.628	0.364
SS	33 ± 11°	35 ± 9°	1.074	0.071

Note: TJK, thoracolumbar junction; LL, lumbar lordosis; SS, sacral slope.

### The equation relationship and error between x-ray and MRI

**Figure 2:**
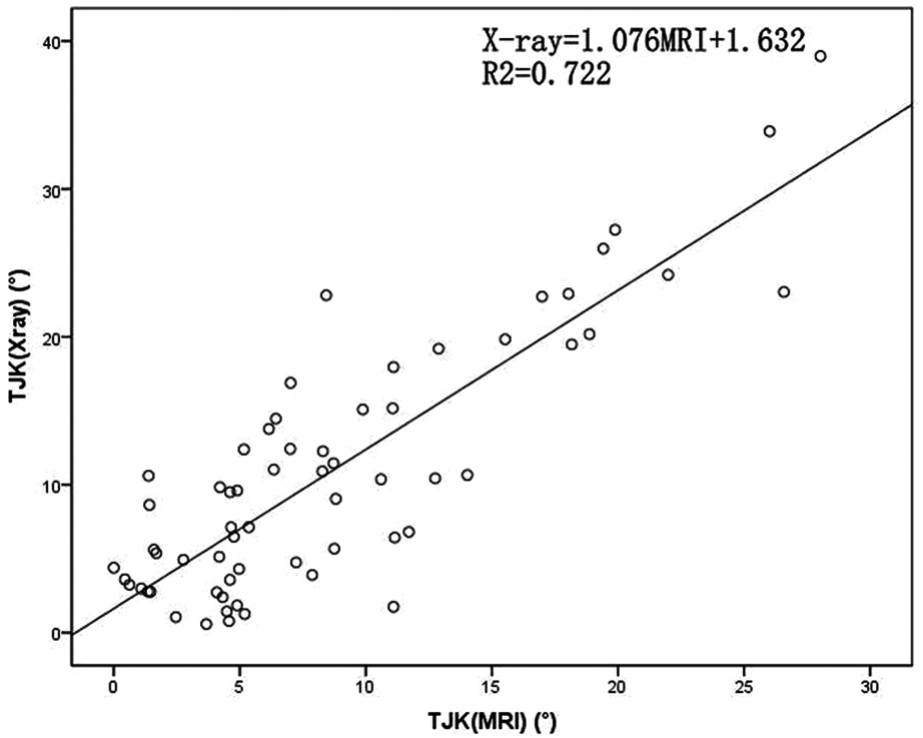
Scatterplot of the thoracolumbar junction kyphosis (TJK) angles determined using plain radiographs and magnetic resonance imaging (MRI). TJK, Thoracolumbar junction kyphosis; MRI, magnetic resonance imaging.

**Figure 3:**
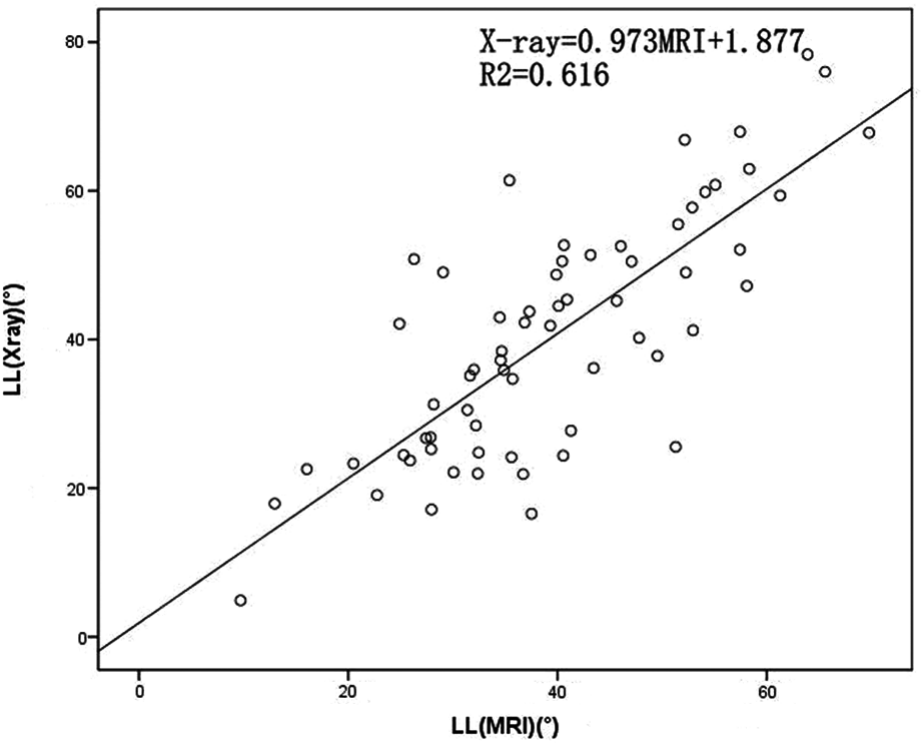
Scatterplot of the lumbar lordosis (LL) measured using plain radiographs and magnetic resonance imaging (MRI). LL, lumbar lordosis; MRI, magnetic resonance imaging.

**Figure 4:**
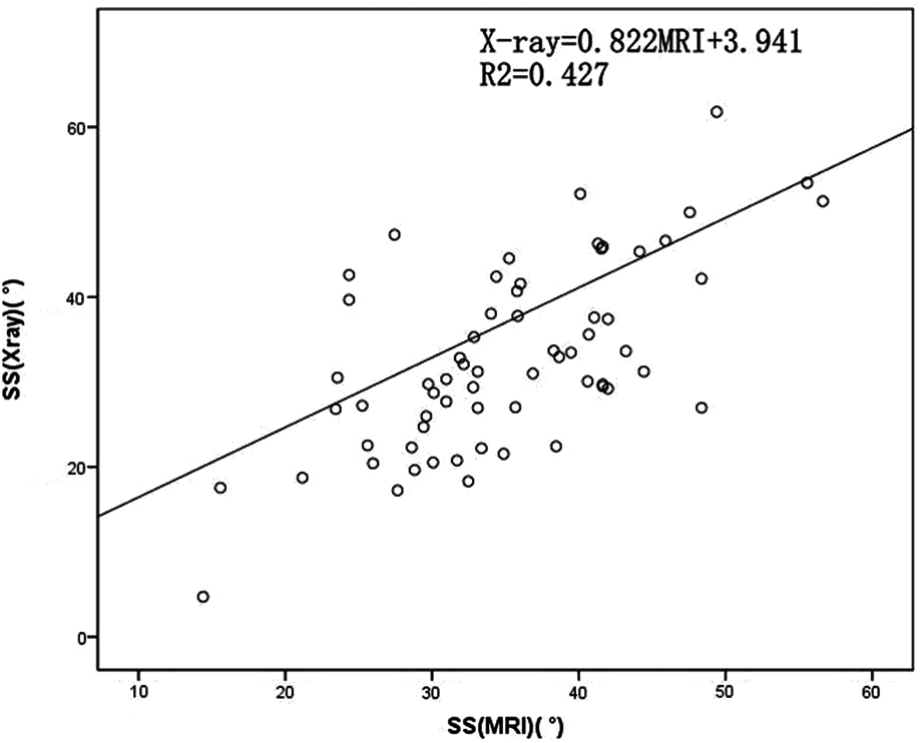
Scatterplot of the sacral slope (SS) measured using plain radiographs and magnetic resonance imaging (MRI). SS, sacral slope; MRI, magnetic resonance imaging.

The pairwise mean differences between the two spine surgeons for LL, TJK and SS angle parameters on radiographs were −0.59(5.36)°, 0.93(3.20)° and −0.55(4.05)°, respectively. The pairwise mean differences between the two spine surgeons for LL, TJK and SS angle parameters on MRI were −0.10(3.96)°, 0.34(2.47)° and 0.01(3.43)°, respectively. The pairwise mean differences between the two spine surgeons for all angular parameters on radiographs and MRI images were lower than 2°, which indicated excellent interobserver agreement for all the parameters. The LL measurements were approximately equal with both imaging methods. Evaluating the relationship between the MRI and radiographic measurements, the Pearson coefficient was r = 0.85 for TJK, r = 0.785 for LL and r = 0.654 for SS. The x-ray and MRI measurements showed relatively linear relationships (Table 3; Figures 2–4).

**Table 3:** Trend equations of x-ray and MRI.

	Best-Fit Equation	Absolute Error (°)
TJK	x-ray = 1.076MRI + 1.632	4.575
LL	x-ray = 0.973MRI + 1.877	10.1
SS	x-ray = 0.822MRI + 3.941	8.227

Note: TJK, thoracolumbar junction; LL, lumbar lordosis; SS, sacral slope.

### Intra-observer reliabilities of x-ray and MRI parameters

The intra-observer reliabilities of the TJK, LL and SS measurements obtained from MRI ranged from 0.965 to 0.972, indicating that the results are in strong to near-perfect agreement. In contrast, the intra-observer reliabilities obtained from the x-rays ranged from 0.800 to 0.943 (Table 4). The inter-observer reliability quantified using ICCs was 0.713 to 0.856 for x-rays and 0.891 to 0.925 for MRI, suggesting strong agreement (Table 5).

**Table 4:** Intraobserver reliability for the 2 observers.

	Observer1		Observer2		Overall	
	x-ray	MRI	x-ray	MRI	x-ray	MRI
TJK	0.956	0.968	0.923	0.971	0.943	0.970
LL	0.856	0.972	0.869	0.952	0.860	0.965
SS	0.815	0.961	0.773	0.978	0.800	0.972

Note: TJK, thoracolumbar junction; LL, lumbar lordosis; SS, sacral slope; MRI, magnetic resonance imaging.

**Table 5:** Interobserver reliability of the measurements from standing x-ray and supine MRI.

	TJK	LL	SS
x-ray	0.782	0.856	0.713
MRI	0.891	0.925	0.910

Note: TJK, thoracolumbar junction; LL, lumbar lordosis; SS, sacral slope; MRI, magnetic resonance imaging.

### The cost and examination preparation time of x-ray and MRI

From the perspective of cost analysis, it is found that the cost of MRI examination is 800 yuan and the cost of x-ray examination is 120 yuan. Regarding examination preparation time, MRI examination takes 8 min, which is significantly longer than the 1–2 min required for x-ray examination (Table 6).

**Table 6:** The cost and preparation time of the two inspections.

Type	Cost (RMB)	Check preparation time (minutes)
MRI	800	10
x-Ray	120	1–2

Note: MRI, magnetic resonance imaging.

## Discussion

Degenerative lesions of the lumbar spine can result in a series of pathological manifestations, such as bone hyperplasia in the lumbar spine, disc herniation, ligament hypertrophy and calcification (17). The common causes are primarily long-term sitting, poor posture, excessive weight-bearing, trauma, cold temperature, etc. For some patients who often experience the above-mentioned predisposing factors, degenerative changes in the lumbar spine occur much more quickly than normal and can cause a series of clinical symptoms, such as lower back pain, lumbar stiffness, limited activity, lower limb radiation pain, numbness, weakness and so a series of performance (18).

The standing x-ray, which spine surgeons use to plan surgery, is considered the gold standard for measuring spinal alignment (19). However, repeated doses of ionising radiation increase the risk of health problems, such as an altered immune response and increased cancer risk (11, 20). Although improvements in radiographic technology have minimised radiation exposure, long-term health complications remain problematic (21, 22). Thus, methods that do not use radiation are needed to evaluate spinal alignment. Although MRIs have lower radiation intensity, they are more costly compared to x-rays and require more preparation time.

In this study, we found that the correlation between the standing radiographic and supine MRI measurements of SS, TJK and LL in DLD were very good. Several studies have explored the relationship between Cobb's angle measured from standing radiographs and supine MRI. Wang et al. (12) used an axial load device to adjust for the unloaded spinal condition present in supine MRI images and found a near-linear relationship between the plain radiographic and MRI Cobb measurements. Some studies also found a near-linear relationship without additional axial loading, although supine MRI tended to underestimate plain radiographs by 10° on average in adolescent idiopathic scoliosis (13, 23). For sagittal balance, Li et al. (24) observed no significant difference between thoracic kyphosis angle measurements from plain radiographs and MRI. Nevertheless, the authors pointed out that these results cannot be transferred to LL measurement. Baldairon et al. (14) compared supine MR imaging and standing x-rays in the evaluation of the sagittal alignment of the upper thoracic spine and found no significant difference in the sagittal angles. However, all the previous studies were performed in patients with adolescent idiopathic scoliosis. In addition, the efficacy of x-ray and MRI in evaluating spinal sagittal alignment in DLD is controversial. Benditz et al. (15) compared MRI with conventional radiography in the assessment of LL, and their study showed that supine MRI can be used to assess LL. In a recent study (25), it was found that supine MRI underestimated the measurements of lumbopelvic sagittal alignment parameters in standing radiographs and recommended that all patients have additional lumbar x-ray examinations in the standing position before surgery.

This study found that the measurements of SS, TJK and LL from supine MRI were strongly correlated with those from standing x-rays (r = 0.85 for TJK, r = 0.785 for LL and r = 0.654 for SS). The coefficient for SS indicated a moderate correlation, which may be due to the poor visibility of the S1 vertebra in standing x-rays (22). The LL measurements obtained from MRI and x-rays were approximately equal. In DLD, the LL is important; when it is significantly reduced, sagittal plane imbalance occurs, leading to the development of flat back syndrome, which is characterised by back pain, stooped posture and an impaired gait (23). Therefore, the restoration of LL is important in the preoperative plan. According to the results of this study, supine MRI can be used in the evaluation of spinal sagittal alignment without unnecessary exposure to radiation.

The S1 vertebra is frequently obscured on x-rays by the overlying ilium; thus, more than 50% of the S1 vertebrae cannot be viewed properly on x-rays (26). The ICC for intra-observer reliability increased from 0.800 (standing x-ray) to 0.972 (MRI) because MRI provided a clearer picture of the vertebrae. Therefore, MRI measurements enable more precise sagittal measurements.

The treatment methods of DLD include drug therapy, physical therapy and many surgical treatments (27). Comprehensive clinical symptoms, spondylolisthesis, spinal stenosis degree and disc herniation are the important bases for deciding whether to choose surgical methods. Not only can MRI accurately identify the type and degree of spondylolisthesis but it can also clearly display spinal stenosis, disc herniation and foraminal nerve root compression (28). It has a short scanning time and consistent scanning parameters and can simultaneously obtain T1, T2 and PD values of tissues. It can also reconstruct weighted images for multiple image comparison, and the contrast between different tissues is better than that of conventional MRI sequences (28). Therefore, MRI examination should be preferred for patients with soft tissue changes such as intervertebral discs and paravertebral muscles.

There are some limitations in this study. First, it primarily evaluates the changes of paraspinal muscle, intervertebral disc and vertebral body through the quantitative parameter values of imaging and lacks the corresponding direct histological and pathological evidence. Second, the small sample size of the study may affect the experimental data. Third, some potential influencing factors have not been analysed in our study. For example, included in the study were patients with spondylolisthesis, where the sagittal arrangement of the spine is greatly affected by gravity and therefore impacted by standing and supine positions. Moreover, because MRI does not include the femoral heads in the scan field, the calculations of the pelvic incidence angle and pelvic tilt are hampered, which are important parameters for evaluating the lumbopelvic sagittal alignment. Finally, we did not consider the impact of patients' own factors on the spine alignment when selecting samples. For example, patients with higher BMI may change the spine alignment. In the future, Meyerding classification can be used to further study the changes of lateral muscles, intervertebral discs and vertebral bodies in patients with different degrees of DLD by expanding the sample size and controlling the influence of contributing factors.

## Conclusion

In conclusion, supine MRI can be directly translated into sagittal alignment angle measurements obtained from standing x-rays with an acceptable degree of accuracy. The use of MRI avoids the impaired visibility caused by the overlapping ilium and reduces the patient's exposure to radiation.

## Data Availability

The original contributions presented in the study are included in the article/Supplementary Material, further inquiries can be directed to the corresponding author/s.

## References

[1] Ravindra VM Senglaub SS Rattani A Dewan MC Härtl R Bisson E Degenerative lumbar spine disease: estimating global incidence and worldwide volume. Global Spine J. (2018) 8(8):784–94. 10.1177/2192568218770769 30560029 PMC6293435

[2] Yasuhara T Sasada S Date I. Lumbar degenerative disease:key for diagnosis. No Shinkei Geka. (2021) 49(6):1233–45, (in Japanese). 10.11477/mf.1436204510 34879343

[3] Diebo BG Henry J Lafage V Berjano P. Sagittal deformities of the spine: factors influencing the outcomes and complications. Eur Spine J. (2015) 24(Suppl 1):S3–15. 10.1007/s00586-014-3653-8 25387425

[4] Youn YH Cho KJ Na Y Kim JS. Global sagittal alignment and clinical outcomes after 1–3 short-segment lumbar fusion in degenerative spinal diseases. Asian Spine J. (2021) 16(4):551–9. 10.31616/asj.2021.0182 34551501 PMC9441428

[5] Zhang Y Mandelli F Mündermann A Nüesch C Kovacs B Schären S Association between fatty infiltration of paraspinal muscle, sagittal spinopelvic alignment and stenosis grade in patients with degenerative lumbar spinal stenosis. N Am Spine Soc J. (2021) 5:100054. 10.1016/j.xnsj.2021.100054 35141619 PMC8820068

[6] Li J Zhang D Shen Y Qi X. Lumbar degenerative disease after oblique lateral interbody fusion: sagittal spinopelvic alignment and its impact on low back pain. J Orthop Surg Res. (2020) 15(1):326. 10.1186/s13018-020-01837-w 32795374 PMC7427743

[7] Oikonomidis S Meyer C Scheyerer MJ Grevenstein D Eysel P Bredow J. Lumbar spinal fusion of low-grade degenerative spondylolisthesis (meyerding grade I and II): do reduction and correction of the radiological sagittal parameters correlate with better clinical outcome? Arch Orthop Trauma Surg. (2020) 140(9):1155–62. 10.1007/s00402-019-03282-9 31734732

[8] Kim HJ Yang JH Chang DG Suh SW Jo H Kim SI Impact of preoperative total knee arthroplasty on radiological and clinical outcomes of spinal fusion for concurrent knee osteoarthritis and degenerative lumbar spinal diseases. J Clin Med. (2021) 10(19):4475. 10.3390/jcm10194475 34640493 PMC8509257

[9] Lingyu L Xuanwen L. Relationship between X—ray, MRI lumbar parameters and prognosis in patients with lumbar spinal stenosis. Photog Sci Photochem. (2022) 40(05):1108–12. 10.7517/issn.1674-0475.220305

[10] Wang M Xu L Chen X Zhou Q Du C Yang B Optimal reconstruction of sagittal alignment according to global alignment and proportion score can reduce adjacent segment degeneration after lumbar fusion. Spine. (2021) 46(4):E257–66. 10.1097/BRS.0000000000003761 33475277

[11] Sun Z Zhou S Wang W Zou D Li W. Differences in standing and sitting spinopelvic sagittal alignment for patients with posterior lumbar fusion: important considerations for the changes of unfused adjacent segments lordosis. BMC Musculoskelet Disord. (2020) 21(1):760. 10.1186/s12891-020-03777-2 33208130 PMC7677842

[12] Baldairon F Charles YP Eichler D Ntilikina Y Sauleau EA Steib JP. Analysis of factors associated with sagittal alignment deterioration after correction of degenerative scoliosis by in situ contouring. Orthop Traumatol Surg Res. (2021) 107(7):103023. 10.1016/j.otsr.2021.103023 34332144

[13] Benditz A Boluki D Weber M Zeman F Grifka J Völlner F. Comparison of lumbar lordosis in lateral radiographs in standing position with supine MR imaging in consideration of the sacral slope. Rofo. (2017) 189(3):233–9, English. 10.1055/s-0042-120112 28002853

[14] Xu C Yin M Mo W. Correlation and differences in lumbopelvic sagittal alignment parameters between lumbar radiographs and magnetic resonance images. Global Spine J. (2022) 12(1):79–84. 10.1177/2192568220947049 32762375 PMC8965307

[15] Meyers AJ Wick JB Rodnoi P Khan A Klineberg EO. Does L5-S1 anterior lumbar interbody fusion improve sagittal alignment or fusion rates in long segment fusion for adult spinal deformity? Global Spine J. (2021) 11(5):697–703. 10.1177/2192568220921833 32875903 PMC8165926

[16] Kim WJ Shin HM Lee JS Song DG Lee JW Chang SH Sarcopenia and back muscle degeneration as risk factors for degenerative adult spinal deformity with sagittal imbalance and degenerative spinal disease: a comparative study. World Neurosurg. (2021) 148:e547–55. 10.1016/j.wneu.2021.01.053 33497826

[17] Wang Z Tian Y Li C Li D Ibrahim Y Yuan S Radiographic risk factors for degenerative lumbar spondylolisthesis: a comparison with healthy control subjects. Front Surg. (2022) 9:956696. 10.3389/fsurg.2022.956696 36311947 PMC9614147

[18] Lippi L de Sire A Desilvestri M Baricich A Barbanera A Cattalani A Can scoliosis lead to spinal cord ischaemia? Early diagnosis and rehabilitation: a paradigmatic case report and literature review. J Back Musculoskelet Rehabil. (2021) 34(1):43–7. 10.3233/BMR-200070 33164924

[19] Hasegawa K Okamoto M Hatsushikano S Shimoda H Sato Y Watanabe K. Etiology and clinical manifestations of double-level versus single-level lumbar degenerative spondylolisthesis. J Orthop Sci. (2020) 25(5):812–9. 10.1016/j.jos.2019.11.004 31839389

[20] Lord EL Ayres E Woo D Vasquez-Montes D Parekh Y Jain D The impact of global alignment and proportion score and bracing on proximal junctional kyphosis in adult spinal deformity. Global Spine J. (2021):21925682211001812. 10.1177/21925682211001812 33977791 PMC10240591

[21] Li R Shao X Li X Liu Y Jiang W. Comparison of clinical outcomes and spino-pelvic sagittal balance in degenerative lumbar spondylolisthesis: minimally invasive oblique lumbar interbody fusion (OLIF) versus transforaminal lumbar interbody fusion (TLIF). Medicine. (2021) 100(3):e23783. 10.1097/MD.0000000000023783 33545942 PMC7837863

[22] Ahlquist S Thommen R Park HY Sheppard W James K Lord E Implications of sagittal alignment and complication profile with stand-alone anterior lumbar interbody fusion versus anterior posterior lumbar fusion. J Spine Surg. (2020) 6(4):659–69. 10.21037/jss-20-595 33447668 PMC7797796

[23] Paolucci T Agostini F Mangone M Bernetti A Cordiani B Bellomo RG Sagittal spine alignment and postural balance in pre-puberty age: a multidisciplinary and multi-professional rehabilitative point of view. J Biol Regul Homeost Agents. (2021) 35(1):367–72. 10.23812/20-621-L 33511839

[24] Chen Q. Application of X—ray, CT and MRI in diagnosis of facet joint degeneration of lower lumbar. Modern Medical Imageology. (2020) 29(11):2085–7.

[25] Fors M Enthoven P Abbott A Öberg B. Effects of pre-surgery physiotherapy on walking ability and lower extremity strength in patients with degenerative lumbar spine disorder: secondary outcomes of the PREPARE randomised controlled trial. BMC Musculoskelet Disord. (2019) 20(1):468. 10.1186/s12891-019-2850-3 31651299 PMC6813060

[26] Zárate-Kalfópulos B Reyes-Tarrago F Navarro-Aceves LA García-Ramos CL Reyes-Sánchez AA Alpízar-Aguirre A Characteristics of spinopelvic sagittal alignment in lumbar degenerative disease. World Neurosurg. (2019) 126:e417–21. 10.1016/j.wneu.2019.02.067 30822583

[27] Lacroix M Nguyen C Burns R Laporte A Rannou F Feydy A. Degenerative lumbar spine disease: imaging and biomechanics. Semin Musculoskelet Radiol. (2022) 26(4):424–38. 10.1055/s-0042-1748912 36103885

[28] Kovacs FM Arana E. Degenerative disease of the lumbar spine. Radiologia. (2016) 58(Suppl 1):26–34. English, Spanish. 10.1016/j.rx.2015.12.004 26872873

